# Selection of Abdominal Cases for Operation

**Published:** 1918-08

**Authors:** 


***The Selection of Abdominal Cases for Operation.*** Owen Richards. *British Medical Journal,* April 27, 1918.

The real failures are the cases in which the patient is wrongly classed as moribund when operation might have saved his life, and those in which a penetrating wound of the abdomen is not recognized as such until it is too late.

Difficulty lies in determining the circumstances under which operation ceases as a rule to be worth while, even though it may yield to occasional success. The decision as to whether a man wounded in the abdomen should be operated on or not is usually based on the time which has elapsed since the injury and upon the rate and character of the pulse.

The question is: what period of delay renders operation as a rule unprofitable? If we assume that wounds which open the stomach or intestine are necessarily fatal, if untreated, and that hemorrhage is not, the proportion of profitable operations in this series to those which were nearly successful is :

| | |
|---|---|
| In the first twelve hours . . . . . . . . . . . . . . | 4 to 1 |
| " " second " " . . . . . . . . . . . . . . | 2 to 1 |
| Later . . . . . . . . . . . . . . . . . . . . . . . . . . | 1 to 3 |

It is impossible to lay down any rule, but roughly, it seems that operation is very profitable in the first twelve hours.

Of the patients who survive operation performed in the first twelve hours a high proportion will have had their lives saved by it, and this is true in a lesser degree of those operated on in the second twelve hours. After this time, most of those who survive operation are those whose injuries were not originally fatal.

Men with a pulse of 120, or over, have less than half the chance of survival which men with a lower pulse have.

Recently wounded men, with a rapid pulse, should nevertheless be operated on, provided that their condition is as good as it is ever likely to be, that they have a reasonable prospect of surviving the actual operation, and that the time taken does not prevent the proper treatment of other wounded

If care is taken to exclude those who have been wounded too long, and those whose condition is too bad, there still remains the danger of failing to include those who have scarcely any symptoms. Every wound of the abdominal wall, however slight, should be explored, whether signs of intestinal injury are present or not. If it is found to be penetrating, the abdomen should be opened.

The only other difficulty which arises in selection is to balance the claims of abdominal cases against those of other wounded in busy times. The necessity for deciding between these claims can be best avoided by good theatre organization, the provision of two tables for each surgeon, and by not allowing slow operators to do abdominal work. A competent surgeon, with a good nurse helping him, can open the abdomen, search the small and large intestines, and close it, in twenty minutes. Ordinary repairs will extend this time to half an hour.

If these measures are insufficient the decision should be left in the hands of the surgeon in charge of the pre-operation ward.

